# Vimentin Levels and Serine 71 Phosphorylation in the Control of Cell-Matrix Adhesions, Migration Speed, and Shape of Transformed Human Fibroblasts

**DOI:** 10.3390/cells6010002

**Published:** 2017-01-22

**Authors:** Emmanuel Terriac, Giovanna Coceano, Zahra Mavajian, Tijmen A. G. Hageman, Andreas F. Christ, Ilaria Testa, Franziska Lautenschläger, Annica K. B. Gad

**Affiliations:** 1 Department of Physics, University of the Saarland, 66123 Saarbrücken, Germany; emmanuel.terriac@physik.uni-saarland.de (E.T.); andreas.f.christ@gmail.com (A.F.C.); f.lautenschlaeger@physik.uni-saarland.de (F.L.); 2 INM – Leibniz Institute for New Materials, 66123 Saarbrücken, Germany; 3 Department of Applied Physics, KTH Royal Institute of Technology, 100 44 Stockholm, Sweden; giovanna.coceano@scilifelab.se (G.C.); ilaria.testa@scilifelab.se (I.T.); 4 Science for Life Laboratory, Division of Translational Medicine and Chemical Biology, 171 65 Stockholm, Sweden; Mavajian@kth.se; 5 Department of Medical Biochemistry and Biophysics, Karolinska Institutet, 171 77 Stockholm, Sweden; 6 KIST Europe, 66123 Saarbrücken, Germany; t.hageman@kist-europe.de

**Keywords:** focal adhesions, vimentin, cell migration, total internal reflection (TIRF) microscopy, stimulated emission depletion (STED) microscopy

## Abstract

Metastasizing tumor cells show increased expression of the intermediate filament (IF) protein vimentin, which has been used to diagnose invasive tumors for decades. Recent observations indicate that vimentin is not only a passive marker for carcinoma, but may also induce tumor cell invasion. To clarify how vimentin IFs control cell adhesions and migration, we analyzed the nanoscale (30–50 nm) spatial organization of vimentin IFs and cell-matrix adhesions in metastatic fibroblast cells, using three-color stimulated emission depletion (STED) microscopy. We also studied whether wild-type and phospho-deficient or -mimicking mutants of vimentin changed the size and lifetime of focal adhesions (FAs), cell shape, and cell migration, using live-cell total internal reflection imaging and confocal microscopy. We observed that vimentin exists in fragments of different lengths. Short fragments were mostly the size of a unit-length filament and were mainly localized close to small cell-matrix adhesions. Long vimentin filaments were found in the proximity of large FAs. Vimentin expression in these cells caused a reduction in FAs size and an elongated cell shape, but did not affect FA lifetime, or the speed or directionality of cell migration. Expression of a phospho-mimicking mutant (S71D) of vimentin increased the speed of cell migration. Taken together, our results suggest that in highly migratory, transformed mesenchymal cells, vimentin levels control the cell shape and FA size, but not cell migration, which instead is linked to the phosphorylation status of S71 vimentin. These observations are consistent with the possibility that not only levels, but also the assembly status of vimentin control cell migration.

## 1. Introduction

Metastasizing tumor cells show increased expression of the IF protein vimentin, which, therefore, has been used to diagnose invasive tumors in the clinic for decades, and is used as a canonical marker of epithelial-to-mesenchymal transition. Recent findings indicate that vimentin is not only a passive marker of carcinoma, but may also induce changes in cell shape and adhesion, as well as cell invasion of tumor cells [1,2,3,4,5].

Cell migration and invasion are governed by the interaction of cells with the extracellular matrix. In many cell types, these cell-matrix adhesions are specific multi-protein entities called focal contacts (FCs) or focal adhesions (FAs). The molecules of cell-matrix adhesions span the cell membrane and physically connect the extracellular matrix with the intracellular cytoskeleton. In particular, the actin microfilament system and microtubules are both known to interact with, and to regulate, the structure and function of cell-matrix adhesions. Vimentin IFs were shown to be linked to cell-matrix adhesions decades ago [6]. Vimentin is important for the transfer of mechanical force from the cell adhesions to the cell interior, and vimentin protein levels regulate cell adhesion strength of endothelial cells [7,8,9,10]. To date, the functional role of the vimentin IF network in the formation and function of cell-extracellular matrix adhesions remains unclear.

Several studies suggest that not only the level of the vimentin protein, but also the assembly status of the vimentin filaments regulates cell adhesion, migration, and invasion [11,12,13]. The assembly of cytoplasmic IFs into filaments is a step-wise process in which the monomer IFs first associate into dimers that then assemble into tetramers, forming unit-length filaments (ULFs). ULFs can then assemble into intermediate filaments [14]. Vimentin has a large number of phosphorylation sites, which control the assembly status and the architecture of the filaments. Phosphorylation of most sites results in the disassembly of filaments, as well as a bundled assembly status [15,16,17,18]. Recent findings by Hyder et al. indicate that sphingolipids can stimulate ROCK-dependent phosphorylation of vimentin specifically at serine 71 (S71), and thereby stabilize the vimentin filaments and inhibit cell motility [17,19]. In contrast, the phosphorylation of this particular site has also been reported to cause the inhibition of IF formation during cytokinesis, and to increase the soluble, non-filamentous fraction of vimentin [20]. The contrasting reports on S71 phosphorylation on vimentin polymerization and its role in processes such as migration and cytoskinesis suggest that the function of S71 is highly complex and that vimentin may be able to react differently depending on the surrounding conditions or in combination with different partners. Such a system may be important where the cytoskeleton needs to be under strict spatial control, allowing separate, and even opposite, signaling cascades to be active in well-defined local areas adjacent to each other.

Most previous studies have analyzed the effects of overexpression of vimentin in cells of endothelial origin, which lack endogenous expression of vimentin. In contrast, in our study, we have used transformed, highly migratory, metastasizing human fibroblasts, which have a high endogenous level of vimentin. These cells have been well characterized, both due to quantitative analysis of the global transcriptome within these cells, and because the levels and localization of many proteins, as well as the cytoskeletal organization and behavior of the cells, have been described previously [21,22,23]. We also previously showed that these cells have reduced FA size, altered spatial distribution of cell-matrix adhesions, and a more entangled vimentin network compared to normal, primary fibroblast [23]. The size of FAs has been shown to be strongly linked to the speed of cell migration, with an optimal size for maximal cell speed of 0.5–1.0 µm^2^ [24]. We speculated that the cell-matrix adhesions of our transformed and invasive cells would be optimal for cell migration, and that overexpression of vimentin in this cell system could possibly revert the transformed cell phenotypes to normal.

To clarify the role of vimentin in the control of cell-matrix-adhesions and cell migration in these transformed fibroblasts, we analyzed the nanoscale spatial organization of vimentin, actin microfilaments, and cell-matrix adhesions using three-color stimulated emission depletion (STED) microscopy. These images were quantified by computational analysis. We further assessed the functional role of vimentin in the control of cell-matrix adhesions and cell migration. Finally, we analyzed how the expression of wild-type and phospho-mutant vimentin regulated FAs, cell shape, and cell migration.

## 2. Results

### 2.1. The Nanoscale Organizations of Vimentin and Cell-Matrix Adhesions Are Linked

**Figure 1:**
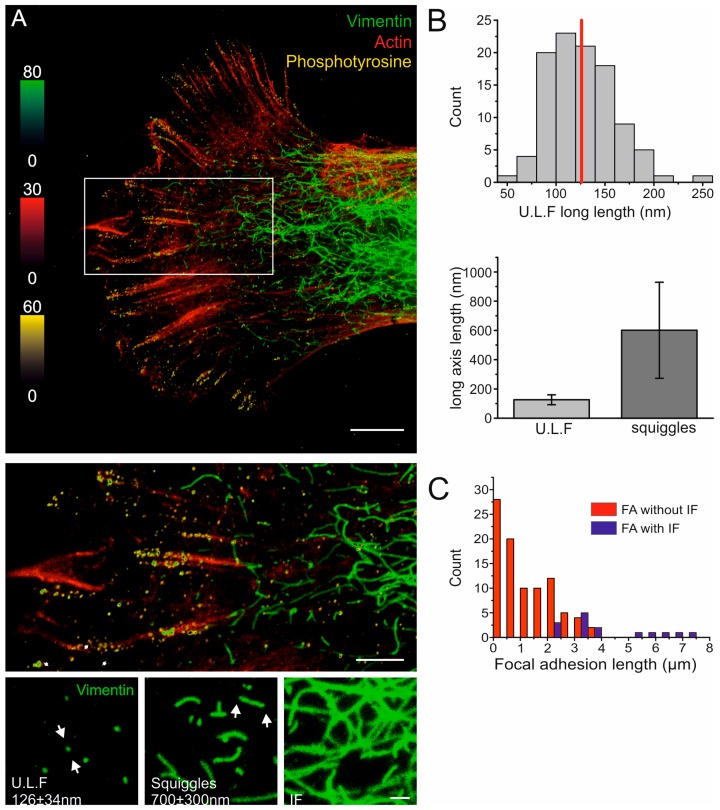
The nanoscale spatial distribution of vimentin is linked to cell-matrix adhesions. (**A**) Top image: three-color STED image of a representative cell expressing wild-type vimentin fused to an EGFP variant and stained for GFP (green), phosphotyrosine (yellow), and actin (red). Scale bar; 5 µm. Magnification of the area marked with a white box in the top image is shown in the middle panel. Scale bar; 2 µm. Lower panel: zoomed images of the assembly states of vimentin filaments, from single ULFs to mature filaments in different regions of the image. Scale bar; 500 nm. The experiment was performed three times, and representative images of a total of 13 STED images are shown; (**B**) top panel: distribution histogram of the measured ULF vimentin lengths, with a vertical red line indicating the median. Below: bar plot showing the lengths of ULFs and squiggles along their long axis; and (**C**) distribution histogram of FA lengths in close spatial association with the presence (blue) or absence (red) of vimentin filaments.

Several studies have indicated that vimentin can control the adhesion of cells to the underlying substrate [7,8,9,10]. We, therefore, aimed to determine the spatial co-distribution of vimentin and FAs at the nanoscale level. To this end, we analyzed the spatial distribution of vimentin, phosphotyrosine, and filamentous actin (F-actin) at the leading edge of cells using STED microscopy, as described in the Method section. When analyzing the extent of co-localization between the phosphotyrosine signal and the vimentin signal in the images, we detected phosphotyrosine-positive dots both in areas with and without vimentin signals in the micrometer range. Structured phosphotyrosine signals, such as FCs and FAs, were observed mainly in the vicinity of a vimentin signal. Occasionally, we observed FCs and FAs in areas with no vimentin signal in the micrometer range (Figure 1A). In contrast, vimentin dots were only found in the proximity (nanometer range) of phosphotyrosine dots, FCs, and FAs. Structured vimentin signals, i.e., squiggles and filaments, were always found close to a structured phophotyrosine signal of FC or FA (Figure 1A). While we very rarely observed an exact overlap between the phosphotyrosine and vimentin dots, we often observed an overlap between phosphotyrosine dots and vimentin squiggles or long filaments (Figure 1A). We found that, in the analyzed part of the cell, vimentin was present at different lengths (Figure 1). When quantifying the length of the vimentin signal, we found that the most common length of vimentin short filaments was 126 ± 34 nm (Figure 1B,C). ULFs have been reported to be approximately 65 nm long [25]. However, together with the sizes of the primary and secondary antibodies and living-color tags used for detection of the ULFs, and the resolution of the microscope, the expected length of an ULF in our system would be 130 nm. These vimentin-positive structures were found to be elongated (Figure S1), further supporting the conclusion that they may be ULFs. The 200-nm- to 1000-nm-long vimentin structures showed an average length of 600 ± 300 nm, and we speculate that this fraction corresponds to vimentin squiggles (Figure 1B,D). We could further detect that the very long and continuous vimentin filaments were spatially localized in the vicinity of large FAs, with a length of 4 µm or longer, but not to shorter FAs or FCs (Figure 1E). Taken together, these observations indicate that both the spatial distribution and the polymerization state of vimentin are closely linked to, and dependent on, the organization and size of cell-matrix adhesions.

### 2.2. Vimentin Controls the Size, But Not the Turn-Over, of Cell-Matrix Adhesions in Transformed Fibroblasts

**Figure 2:**
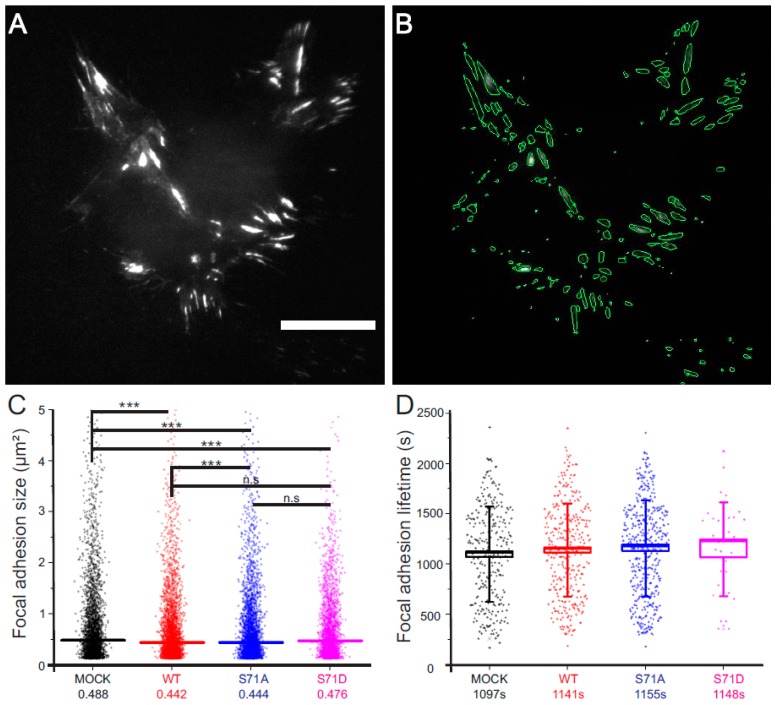
Expression of wild-type, S71-phospho-mimicking or -deficient vimentin changes the size but not the turn-over of cell-matrix adhesion. (**A**) The GFP-Paxilin signal in a representative cell acquired by total internal reflection microscopy and, thereafter, (**B**) computationally processed to show the segmented areas defined as FAs. Scale bar; 20 µm; (**C**) the size of FAs under the different conditions, as indicated. The horizontal colored line corresponds to the median. *** *p* < 0.001 (Student’s *t*-test); and (**D**) lifetimes of the FAs, as indicated. The box corresponds to the standard error of the mean, and the whiskers to the standard deviation. Mean values are indicated by the number under each condition.

To determine whether the protein levels of vimentin or the S71 phosphorylation status of vimentin controls the FAs in our system, we expressed fluorescently tagged vimentin wild-type, or S71 phospho-mimicking or phospho-deficient vimentin in our cells, and analyzed the FA size and lifetime, as described in Figure 2A,B, as well as in the Methods section. We observed that expression of all the vimentin variants, i.e., the wild-type, the phospho-mimicking or the phospho-deficient variant, reduced the size, but not the lifetime, of FAs (Figure 2C,D). Taken together, these data suggest that vimentin protein expression is sufficient to reduce the size of FAs. Consistent with the concept that soluble, non-filamentous vimentin, or short fragments of vimentin, may reduce the size of FAs, we observed that a cell region would show a rapid increase in non-filamentous vimentin just prior to FA dissolution and lamellipodium formation (Figure S2).

### 2.3. Vimentin Protein Levels, But Not Vimentin S71 Phosphorylation, Controls the Shape of Transformed Fibroblasts

**Figure 3:**
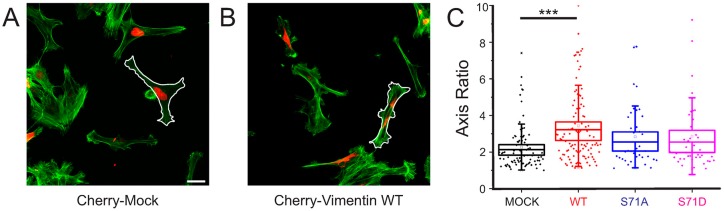
Expression of wild-type but not S71-phospho-mutant vimentin changes the axis ratio of the cells. (**A**,**B**) Representative images of cells expressing (**A**) Cherry-mock or (**B**) Cherry-vimentin, with regard to F-actin (green) and vimentin or control (red). The thin white lines outline the cell borders, as drawn and used for axis ratio analysis. Scale bars; 35 µm; (**C**) Quantification of the axis ratio of cells under the different conditions, as indicated. The boxes correspond to the standard error of the mean, and the whiskers to the standard deviation. *** *p* < 0.001 (Student’s *t*-test).

Increased levels of the vimentin protein are a key marker of epithelial-to-mesenchymal transition and have been found to be both required and sufficient for an elongated phenotype in epithelial cells [26]. To determine whether vimentin causes an elongated phenotype in our cells, we analyzed the length versus the width of cells expressing wild-type or phospho-mutant vimentin, as described in Figure 3A,B and the Methods section. We observed that exogenous expression of the vimentin wild-type protein resulted in a more elongated shape of our cells compared to the control cells (Figure 3). We observed the same phenomenon in the total internal reflection (TIRF) microscopy films of Figure 2. In addition, we used STED microscopy to investigate the fine details in F-actin organization of differently shaped cells (Figure S3). We observed no statistically significant differences in cell shapes when expressing the phospho-mutants (Figure 3C), and no correlation between subtle differences of the levels of ectopically-expressed wild-type vimentin and the elongated shape of cells (data not shown). These results are in line with the previous literature that suggests that major changes in vimentin protein levels control the shape of cells [27].

### 2.4. Vimentin S71 Phosphorylation Increases the Speed of Transformed Fibroblasts

**Figure 4:**
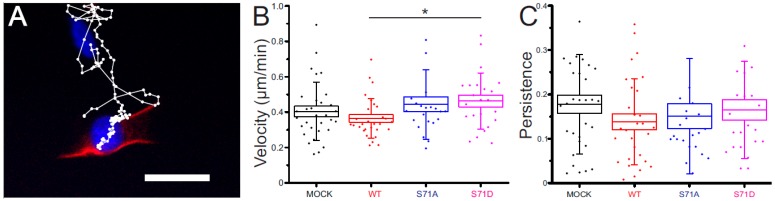
Migration characteristics. (**A**) Overlay of the last frame of a representative movie, with the trajectory of the cell during the movie. Scale bar; 50 µm; (**B**) quantification of the mean instantaneous velocity of cells under the different conditions, as indicated. * *p* < 0.05 (Student’s *t*-test); and (**C**) quantification of the persistence of cells for the trajectories represented in (**B**).

To clarify whether the observed changes in FA size and cell shape induced by vimentin can control cell migration, we expressed the vimentin constructs in cells and analyzed the capacity of the cells to migrate, as described in the Materials and Methods section and shown in Figure 4A. Our results show a significant increase in the migration speed in cells expressing the phospho-mimicking S71 mutant of vimentin (S71D), as compared to vimentin wild-type (Figure 4B). The other constructs did not result in statistically different migration speeds, as compared to wild-type vimentin. We could not detect whether any of the vimentin constructs changed the persistence of the cell migration (Figure 4C). These results suggest that phosphorylation at S71 of vimentin could be sufficient to promote the speed of cell migration.

## 3. Discussion

We found that, at the nanoscale level, vimentin exists in fragments of different lengths, where most short fragments were of the predicted size of ULFs [25]. While the shorter fragments were predominantly present in the vicinity of small cell-matrix adhesions, the longer filaments were mostly found in the proximity of large FAs. We observed that increased levels of vimentin reduced the sizes of FAs, supporting the idea that the association of vimentin to cell-matrix adhesions can regulate the adhesions. However, the vimentin levels did not change the lifetime of FAs. The long filaments of vimentin were found close to cell-matrix adhesions that were significantly larger than the migration-promoting size of adhesions (known to be 0.5–1 µm^2^ ), which supports the idea that long, stable vimentin filaments do not promote, but rather stabilize, adhesions and inhibit cell migration [10]. Can short vimentin units promote cell migration? This idea is supported by several previous observations [11,26]. Consistent with these finding, we observed that a rapid local increase in a diffuse, non-filamentous fraction of vimentin precedes dissolution of FAs and formation of lamellipodia (Figure 2). The most common size of short vimentin filaments that we observed was of the expected size of ULFs, indicating that these short fragments are often ULFs, and that the vimentin in the vicinity of FCs is often as ULFs. These observations are in line with the previous findings suggesting that that short filaments of vimentin could function as a scaffold that recruits the required molecules to the appropriate site; i.e., lamella [28]. Then, when this function is fulfilled, vimentin would no longer be needed to induce migration, but rather to form long filaments that stabilize FAs and inhibit migration [17].

Fibroblasts lose their elongated shape due to their malignant transformation with fibroblasts with oncogenes. Our observation that increased vimentin levels resulted in a more elongated cell shape of transformed cells suggests that vimentin can revert an oncogenically transformed fibroblast morphology into that of normal fibroblasts. This is in line with previous observations in the field that vimentin induces an elongated cell shape [26], and highlights the importance of IFs in the control of cell shape.

In contrast to earlier reports, vimentin protein levels did not regulate the speed or directionality or migration. We speculate that this discrepancy is due to the different cell systems used. Most studies that have observed that vimentin levels control cell migration have used endothelial cells, which often lack endogenous vimentin and are less migratory than our transformed mesenchymal cells. Our cells have a high endogenous level of vimentin and a high capacity to migrate and invade, and we speculate that in this background, more vimentin cannot further stimulate migration. Taken together with our data above, we conclude that the vimentin-induced reduction of FA size and increased elongated shape of cells is not sufficient to induce cell migration in our cell system.

**Figure 5:**
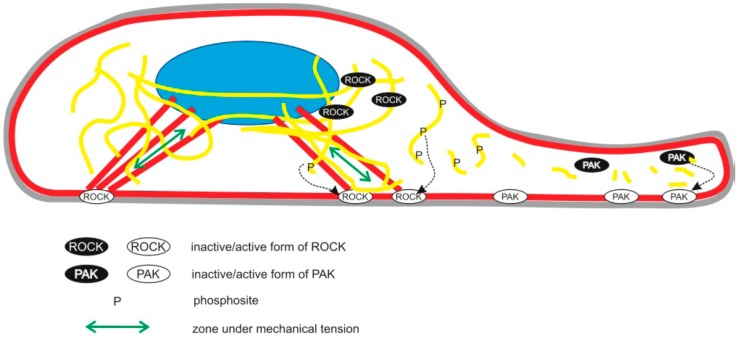
Working hypothesis. Image showing the plasma membrane (grey), nucleus (blue), actin cytoskeleton (red), and vimentin filaments (yellow). The localization of the active and inactive configurations of the ROCK and PAK kinases and S71-phosphorylated vimentin are shown, as indicated. We speculate that at cell regions where the F-actin stress fibers and vimentin filaments are subjected to mechanical load through their linkage to FAs (as indicated with green arrows), the S71 phosphorylation of vimentin filaments would result in lower affinity for ROCK, ROCK translocation to the plasma membrane, and its activation, and an increased mechanical load. We suggest that in cell areas where vimentin is not under mechanical load, S71 phosphorylation would result in severing of vimentin filaments into smaller units, which may stimulate Rac-PAK signaling, and promote vimentin severing and lamellipodium formation. Thereby, the level of the mechanical load would govern which of these two counteracting pathways is stimulated by S71 vimentin phosphorylation.

Similar to cytokinesis, cell migration requires that the cytoskeleton is under strict spatial control, allowing separate—and even opposing—signaling cascades to be active in well-defined local areas in the vicinity to each other. Hence, cell migration is a result of a polarized organization of a contractile cytoskeleton and cell-matrix adhesions, which is a result of a biochemical and mechanical signaling gradient inside the cell. This gradient can be caused by spatially-restricted, local activation of Rac-PAK at the front of the cell, and by the activation of RhoA-ROCK signaling in a more rearward position and at the back of the cell [29]. The antagonistic nature of both pathways may help in their spatial restriction [30,31,32]. To date, the role of vimentin in the control of this gradient remains elusive. We could observe that the phospho-mimicking (S71D) mutant of vimentin increased the speed of cell migration, as compared to wild-type vimentin. We speculate that this is because the phospho-mimicking S71D mutant promotes the gradient that causes cell migration. Recent observations by Komura et al. showed that S71 phosphorylation of vimentin can result in severing of vimentin filaments into tetramers [33], and local depolymerization of vimentin appears to activate Rac-signaling [11]. Therefore, we suggest that S71 phosphorylation of vimentin can result in vimentin depolymerization or cleavage, inducing local Rac-PAK activation, which induces smaller adhesions and lamellipodium formation at the front of the cell (Figure 5).

In 1998, Wun-Chey Sin et al. proposed that a large fraction of inactive ROCK can bind to the vimentin filament network, and that ROCK-dependent phosphorylation of vimentin filaments can release ROCK from the filaments and induce the translocation to the plasma membrane and further activation of ROCK1 [34]. Several studies suggested that vimentin is connected to FAs and that this connection is required for the transfer of mechanical forces from the cell surface integrins to the cell interior [8]. Therefore, we speculate that in regions where actin is under tension (e.g., stress fibers), the stress may be expected to be locally transmitted to the vimentin network. Our data are in line with the hypothesis that in the cell areas where the vimentin filaments are anchored to FAs and exposed to mechanical stress, the S71 phosphorylation of the vimentin network would mainly result in an activation and release of ROCK, reinforcing the local tension in the stress fibers via activation of myosin2 (Figure 5).

In contrast, in subcellular locations where vimentin filaments are not under tension, the S71 phosphorylation would result in severing of the vimentin filaments into shorter fragments or its dissolution into tetramers (Figure 5). This concept is consistent with the observation that in cells with a collapsed vimentin network, vimentin S71 phosphorylation increases the level of short vimentin fragments, and that vimentin filaments are recomposed by severing and reannealing of short fragments [33,35]. As described above, an increase in the available pool of soluble vimentin or short vimentin fragments could activate Rac1 and lamellipodium formation. Our data are, therefore, consistent with the hypothesis that depending on the local subcellular mechanical context, S71 phosphorylation may result in both local cleavage of the filaments into shorter units, promoting Rac-PAK signaling, as well as activation of ROCK-dependent contractile forces, and that the strengthening of both of these antagonistic pathways promotes cell polarization and migration (Figure 5). Nonetheless, it is important to note that many of the ideas presented above are hypothetical and require future experimental validation.

Taken together, our results suggest that in highly migratory, transformed mesenchymal cells, vimentin levels control the cell shape and FA size, but not the speed of cell migration, which, instead, can be linked to the phosphorylation status of S71 vimentin. These observations are consistent with the possibility that not only the levels, but also the polymerization status of vimentin control cell migration.

## 4. Materials and Methods

### 4.1. Cell Culture, DNA Constructs, and Transfection Procedures

The transformed and metastasizing cells were previously constructed by Hahn et al. by inserting three well-defined genetic elements: the telomerase catalytic subunit (*hTERT*) in combination with two oncogenes (the simian virus 40 large-T oncoprotein, and an oncogenic allele of H-*ras*) into neonatal human dermal fibroblasts [22]. The cells were cultured as described previously [36]. These cells have been characterized previously with regard to their total gene transcription and protein expression, as well as the cytoskeletal organization and behavior of the cells [21,22,23]. For confocal and single-color STED experiments, the cells were transiently transfected with a plasmid coding for mCherry fused to the protein of interest, 24 h prior to analysis, as described previously [2]. For three-color STED analysis, the cells were transfected with an EGFP variant fused to wild-type vimentin [37].

### 4.2. Immunofluorescence Staining, Confocal and STED Imaging and Computational Analysis of STED Images

The cells were fixed and permeabilized as described previously [38]. Prior to confocal and single-color STED imaging, the actin in the cells was stained with Phalloidin-Oregon Green488 (Thermofisher, Waltham, MA, USA). For three-color STED imaging, the cells were stained with a rabbit anti-GFP antibody (Abcam, Cambridge, MA, USA), and an Alexa488-conjugated secondary antibody (Abcam), a mouse anti-phosphotyrosine antibody (Santa Cruz Biotechnology, Dallas, TX, USA) and an Atto594-conjugated secondary antibody and with Phalloidin-Alexa647 (Sigma-Aldrich, St. Louis, MO, USA). Confocal images were recorded with a laser-scanning microscope (Zeiss LSM 780) equipped with a 40×/1.2 water immersion objective. The experiments were performed exciting the Phalloidin-Oregon green488 at 488 nm and the mCherry fused to vimentin at 561 nm. A field of view of 353 × 353 µm was chosen, and the fluorescence emission signal of both green and red channels were acquired at the same time. The STED images of actin were recorded with a custom built gated-STED microscope equipped with a 100×/1.4 oil objective, using a 473 nm laser line to excite Phalloidin-Oregon green488 and a 590 nm laser to deplete the signal. The three-color STED imaging was performed on a Leica TCS SP8 3X STED microscope equipped with a 100×/1.4 STED WHITE objective. The images were recorded by exciting the vimentin-Alexa488, Phoshotyrosin-Atto594 and Phalloidine-Alexa647 with 488 nm, 590 nm, and 650 nm laser lines, respectively. We used the STED beam at 590 nm to deplete Alexa488 and at 775 nm to deplete Atto594 and Alexa647. For imaging analysis, we focused on protruding regions of the cells, according to the organization of the actin filament system. For each independent experiment, 3–8 images from different cells were acquired, per cell type per condition. Three independent experiments were performed, yielding a total of 15 images per cell type per condition. The images were acquired with 20 nm pixel size and a pixel dwell time of 80 μs. All acquired or reconstructed images were processed and visualized using the ImSpector software (Max-Planck Innovation, Goettingen, Germany) and ImageJ (https://fiji.sc/, [39]). Brightness and contrast were linearly adjusted for the entire images. We further quantified the size and the spatial distribution of vimentin and FAs in the three-color images. The vimentin–FA quantification was made in the region that was within 10 µm of the cell edge (as defined by the most peripheral phosphotyrosine signal), and the border of the vimentin network, or between the vimentin network and the cell edge. We measured the length and the thickness of vimentin ULFs with a custom-modified ImageJ macro called FWHM_Line: the filament length and thickness were defined as the FWHM of a fitted Gaussian for the one-pixel-wide line profile drawn on the filaments. The same analysis was performed to measure the thickness of the squiggles, while their length was measured with the Segmentation-Neurite tracer plugin of ImageJ, as shown in Figure S1. We used the confocal images to quantify the length of the FAs, and determined the presence of continuous vimentin filaments in the very proximity of the FAs in the STED images using FIJI.

### 4.3. Cell Shape Analysis

To quantify the elongated shape of cells, we captured confocal images using a 20× objective, outlined the borders of the F-actin staining in cells found to express the various vimentin constructs, as shown in Figure 3A,B, and quantified the axis ratio of these areas, using the FIJI program (https://fiji.sc/, [39]). The individual FAs of the single cells were analyzed by time-lapse TIRF microscopy, followed by computational analysis. Experiments were carried out on a Nikon Eclipse TI-E microscope (Nikon GmbH, Düsseldorf, Germany) equipped with a TI-TIRF-E motorized illuminator, to generate the evanescent field. Cells were transfected with GFP-Paxilin and either cherry-Mock, cherry-WT vimentin, Cherry-S71A, or Cherry-S71D vimentin. Only cells fluorescent for the two fluorophores were considered. Images were taken with a 100×/1.49 Apochromat TIRF objective and an Ixon3 DU-897-BV EMCCD camera. Images were recorded every 3 min for 2 h.

### 4.4. Focal Adhesion Size and Lifetime Measurements

FAs were automatically tracked by a custom written MATLAB program (Mathworks Inc., Natick, MA, USA) that separated detection and tracking of the FAs. Images were pre-processed by performing upsampling, followed by low-pass filtering to get rid of high-frequency noise and high-pass filtering to get rid of image gradients. Thresholding then marked the FAs. A watershed algorithm was used to distinguish closely spaced adhesions that were connected in the thresholded image. A skeletonized thresholded Laplacian can define the centers of elongated objects and was, therefore, used to enforce local minima in the seed image for watershedding. The center of mass of the resulting blobs defined the location of the adhesions. Tracking of adhesions was based on a straightforward neighbor search in subsequent images. Detected FAs in subsequent images were considered the same when they were uniquely within a search radius. If no new adhesion was found within the region, the trajectory was ended. If a new adhesion was found, a new trajectory was started. If multiple adhesions were found within the search radius, the trajectory was ended and multiple new ones were started. The fluorescent intensity was recorded over the trajectories and fitted to a Gaussian curve after background subtraction. Fits giving a chi-square of less than 70% were rejected. For the characteristic lifetime for FAs, we considered the full width at half maximum of the fit.

### 4.5. Cell Migration Experiments

The cells were filmed in media containing Hoechst (50 ng/mL) using ImageXpress Micro XLS (Molecular Devices LLC, Sunnyvale, CA, USA) with the enviromental control active (humidity, temperature, CO_2_). Pixel size was 0.325 µm, and the image size was 702 × 702 µm. The images were taken with a 20× objective, binning 1, at 10 min intervals for 24 h. Thereafter, the trajectories were generated using the FIJI (https://fiji.sc/, [39]) “Manual Tracking” plug-in and quantified through the “Chemotaxis Tool” (http://ibidi.com/software/chemotaxis_and_migration_tool/, Ibidi GmbH, Martinsried, Germany) plug in. The quantities used here were the mean values of the instantaneous speed as well as the persistence (called the directionality), and defined as the ratio of the Euclidian length of the trajectory over its total length.

### 4.6. Statistical Analysis and Plots

The statistical analyses were performed using Student’s *t-*tests with two-tailed heteroscedastic variance. Graphs were generated using the OriginPro software (OriginLab Corporation, Northampton, MA, USA). Unless otherwise specified, box plots show the data as points on top of the statistics box. The range of the box depicts the standard error of the mean (s.e.m) and the whiskers represent the standard deviation. Statistical analyses were performed using two samples Student *t*-tests.

## Supplementary Materials

The Supplementary Materials are available online at www.mdpi.com/2073-4409/6/1/2/s1.
