# Supplementary material for: Vimentin Levels and Serine 71 Phosphorylation in the Control of Cell-Matrix Adhesions, Migration Speed, and Shape of Transformed Human Fibroblasts
Source: Cells. 2017 Jan 22;6(1):2. doi: 10.3390/cells6010002 (PMC5371867; doi:10.3390/cells6010002)
Supplement: Supplementary file 1 [file cells-06-00002-s001.pdf]

Supplementary Materials: Vimentin Levels and Serine 71 Phosphorylation in the Control of Cell-Matrix Adhesions, Migration Speed and Shape of Transformed Human Fibroblasts

Emmanuel Terriac, Giovanna Coceano, Zahra Mavajian, Tijmen Hageman, Andreas F. Christ, Ilaria Testa, Franziska Lautenschläger and Annica K. B. Gad

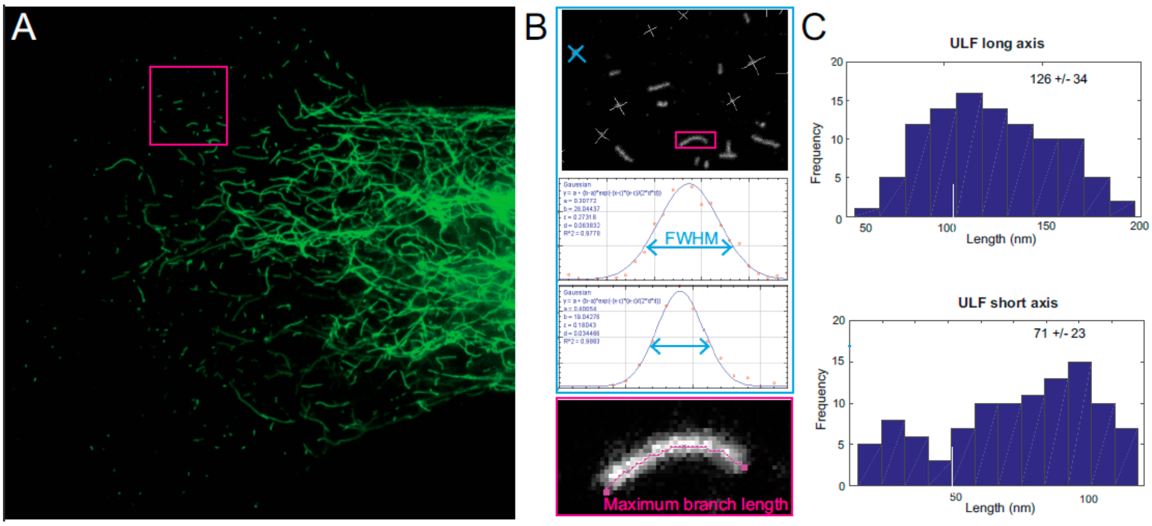

Figure S1. Analysis of the size of the vimentin signal. (**A**) Single color channel of vimentin signal of Figure 1. A magnification of the area indicated with a red square in Figure 1 is shown at the top of the panel (**B**), with line profile measurements drowned for ULFs and squiggles. The Gaussian fit and an example of the branch length measures are shown in the lower panels. (**C**) Distribution histograms of measured ULFs vimentin lengths along both longer (upper) and shorter (lower) axis.

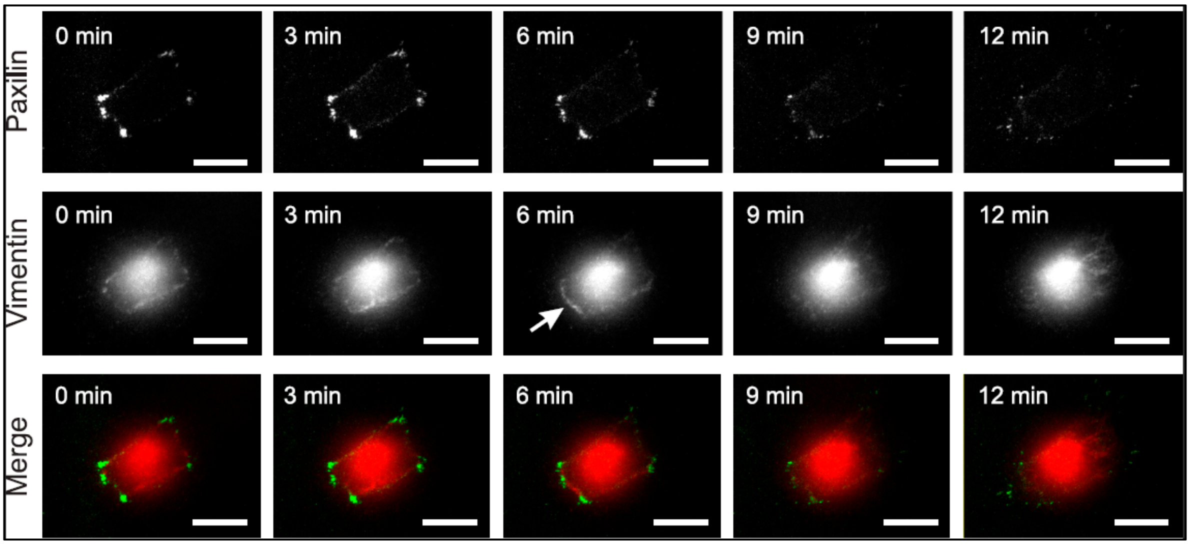

Figure S2. Soluble vimentin s recruited to the cell edge prior to focal adhesion dissolution and lamellipodium extension Montage of 5 sequential pictures (time frame of 3 min) of a movie, recorded by TIRF, of a S71A phosphor-mutant cell. Top row shows the signal for GFP-paxilin, middle row the signal for mcherry-vimentin S71A and bottom row is an overlay of both other (green; paxilin signal, red; vimentin). The arrow indicates soluble vimentin at the cell edge just prior to FA dissassembly. Scale bars are 20 μm.

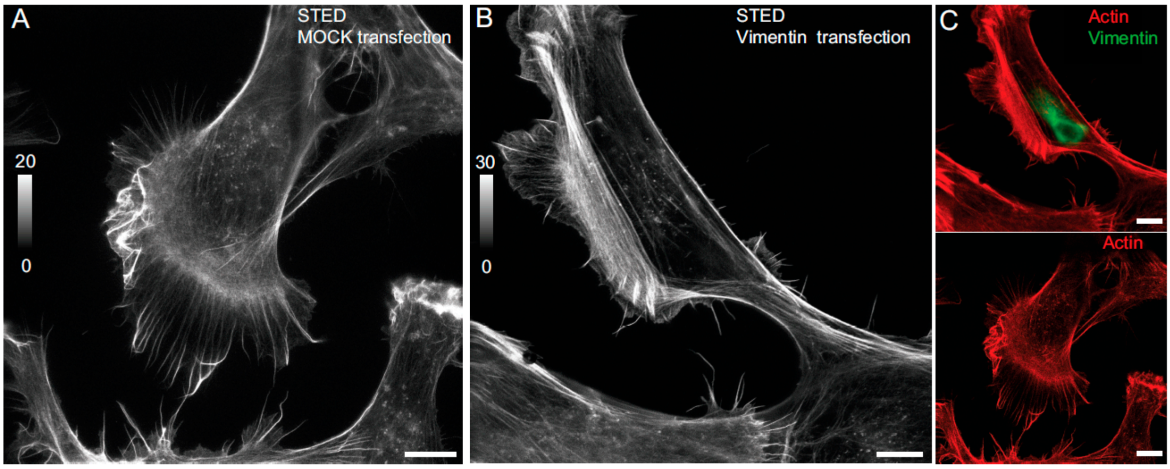

Figure S3. Nanoscale organization of actin in vimentin-overexpressing cells. STED images of actin stained with phalloidin-Oregon green488 for (**A**) Cherry-mock and (**B**) Cherry-vimentin expressing cells. (**C**) Dual color confocal images of the cells in **A** and **B**, showing endogenous actin and exogenous vimentin. Scale bar; 10 µm.
